# Drug repurposing in pharmaceutical industry and its impact on market access: market access implications

**DOI:** 10.3402/jmahp.v2.22814

**Published:** 2014-02-25

**Authors:** Susana Murteira, Aurélie Millier, Mondher Toumi

**Affiliations:** 1 University of Lyon, University Claude Bernard Lyon I, UFR d'Odontologie, 11 rue Guillaume Paradin, 69372 Lyon, Cedex 08, France; 2 Lundbeck SAS, 37-45, Quai du Président Roosevelt, 92445 Issy-les-Moulineaux, Cedex, Paris, France; 3 Creativ-Ceutical S.A., 215, rue du Faubourg St-Honoré 75008 Paris, France

**Keywords:** repurposing, reformulation, repositioning, price, reimbursement, market access

## Abstract

**Background:**

Drug repurposing is a group of development strategies employed in order to overcome some of the hurdles innate to drug research and development. Drug repurposing includes drug repositioning, reformulation and combination.

**Objective:**

This study aimed to identify the determinants of successful market access outcome for drug repurposing in the United States of America (USA) and in Europe.

**Methods:**

The case studies of repurposing strategies were identified through a systematic review of the literature. Price information and reimbursement conditions for all the case studies were collected mainly through access of public datasources. A list of attributes that could be associated with market access outcome (price level and reimbursement conditions) was developed, discussed, and validated by an external expert group. Detailed information for all attributes was researched and collected for each case study. Bivariate regression models were conducted to identify factors associated with price change for all repurposing cases. A similar analysis was performed for reformulation and repositioning cases, in the USA and in Europe, separately. A significance level of 5% was used for all analyses.

**Results:**

A total of 144 repurposing case studies were included in the statistical analysis for evaluation of mean price change. Combination cases (the combination of two or more individual drug components) were excluded from the statistical analysis due to the low number of cases retrieved. The main attributes associated with a significant price increase for overall repurposing cases were ‘change in administration setting to hospital’ (374%, p<0.0001), ‘addressing unmet needs’ (69%, p<0.05), ‘reformulations belonging to Group 3’—that is, change in administration route (117%, p<0.001), and being a repurposed product with the ‘same brand name’ as the original product (65%, p<0.05).

**Conclusion:**

We found that the ability of the repurposed product to address unmet needs, a reformulation where the target product had a different administration route than the source product, and having a similar brand name for repurposed and original products, were variables that impacted a positive price change for repurposed drugs overall. Our research results also suggested that orphan designation could have a positive impact for repositioning in the USA, in particular. Although a change of administration from ambulatory to hospital setting seemed to be significantly correlated with a price increase for the target product, only one case was retrieved for this parameter; as such, it was not possible to infer a firm correlation between this parameter and a change in price.

Several techniques and strategies have been designed by drug developers in order to overcome some of the hurdles innate to drug research and development. Drug repurposing is one such strategy and includes drug repositioning (a new indication in a different therapeutic area, drug reformulation (a change in the formulation) and/or a different pharmacological target), and drug combination (the combination of two or more individual drug components) (1, 2).

This is the third article of a research series focused on evaluating drug repurposing and market access implications. In the two previous articles, we have proposed a harmonized nomenclature and taxonomy for repurposing strategies (1) and have evaluated the regulatory path associated with each type of repurposing strategy (3).

The increase in regulatory requirements for drug development and approval as well as country-specific pricing and health technology assessment (HTA) requirements are considered to have contributed to the reduction of the number of repurposed drugs proposed for development in recent years (3).

Before initiating a drug repurposing process, three major elements of the new drug need to be validated and locked by the developer: regulatory path for the target indication, intellectual property protection, and opportunity for premium price with favorable reimbursement conditions. The first two parameters were evaluated in the second article of this series (3). In terms of opportunity for premium price with favorable reimbursement conditions, an evaluation of the variables possibly associated with this outcome will be conducted in this study.

## Objectives

The aim of this study was to identify the determinants of successful market access outcome for repurposed drugs in the USA and in Europe.

## Methods

The case studies for repurposing strategies that were presented and evaluated in the second article of this series were also used for this study (3). The case studies were identified through a systematic literature review that retrieved 144 repurposed cases, corresponding to a total of 43 active substances.

A review of price and reimbursement conditions at time of the approval of the target product was conducted for all case studies (for source and target product). A list of attributes that could be associated with market access outcome—that is, price level and reimbursement conditions of repurposed drug (target product) versus the originator drug (source product)—was developed and validated by an external expert group. Detailed information for all attributes was researched and collected for each case study. In order to evaluate the association of the different attributes and market access outcome, a statistical methodology was developed.

### Price and reimbursement conditions

Price comparison was conducted using the wholesaler purchasing price (WPP) for Europe case-studies and the wholesale acquisition cost (WAC) for the USA case-studies. This price level was chosen because it does not include margins for wholesalers or pharmacies or value added tax (VAT).

Prices for the source and target product were mainly collected from publicly available sources. All data sources and respective Web links are listed in Supplementary file. For the case-studies from the United Kingdom (UK), we assumed that WPP was discounted 12% from PPP because the pricing source did not list WPP (4).

In order to conduct the statistical analyses, all prices were extracted and used in local currency since price calculation and comparison were country specific.

For each source product, we identified the most recent price available before repurposing; for each target product, we identified the price available immediately after repurposing or as early as possible thereafter.

When the administration route of the target product was the same as the source product, or when the defined daily dose (DDD) was unchanged, we compared the prices for the same strengths and same pack sizes (5). If the same pack size was not available, we compared DDD prices for the closest available pack sizes. When the route of administration was different between source and target product, we compared the DDD using the closest pack size in terms of number of treatment days, and we calculated the price in terms of price per treatment day. The algorithm for price comparison for each repurposing strategy is presented in Table I.

**Table I:** Algorithm for price comparison by repurposing strategy type

Repurposing strategy	Price comparison	Rationale
Repositioning	Price per unit (e.g., mg): - Calculate price per unit (e.g., mg) for the source (‘P/unit A’) and repositioned product (‘P/unit B’) - Then, compare the calculated prices as follows: • Price change=(‘P/unit B’ – ‘P/unit A’)/‘P/unit A’	The added value of a repositioned product lies in the different therapeutic use for the target product. In order to evaluate what exactly is the value attributed to the different therapeutic use of the target product, an assumption of price per mg allows one to offset variations for different doses used (i.e., pharmacodynamics variations).
Reformulations	Price per therapeutic dose (ideally maintenance dose): - For the source (A) and the reformulated product (B), define the therapeutic doses (D1 for A, D2 for B) - Calculate the prices per therapeutic dose (‘P/D1’ for A and ‘P/D2’ for B) - Then, compare the calculated prices as follows: • Price change=(‘P/D2’ – ‘P/D1’)/‘P/D1’	The added value of a reformulation lies mainly is the difference in terms of ADME and/or convenience of administration. Because the therapeutic area for source and target products is the same, the comparison of equivalent therapeutic doses will provide the information regarding the added value considered for the reformulation.
Repositioning and reformulation	Price per equivalent unit dose: - Calculate price per unit (e.g., mg) for the source (‘P/unit A’) and target product (‘P/unit B’) - Define the therapeutic dose for each indication (D1 for A, D2 for B) - Then, calculate the unit price per therapeutic dose for each product (equivalent unit dose) (‘P/equivalent dose’=‘P/unit’ X D) - Finally, compare the calculated prices: • ‘Price change’=(‘P/equivalent dose B’ – ‘P/equivalent dose A’)/‘P/equivalent dose A’	The evaluation of price per unit allows comparing beyond differences in therapeutic doses (i.e., adjusting for differences that may exist in terms of pharmacodynamics). After this, an adjustment is made to compare the price on the basis of the therapeutic doses in order to adjust for differences in ADME, if any.

In this study, we considered price and reimbursement as proxies for market access. Reimbursement conditions were collected through the evaluation of reimbursement databases and HTA evaluation reports. The same sources as those for the collection of pricing data were used and were complemented with a review of additional data sources specific to reimbursement and HTA. All data sources and respective Web links for reimbursement data and HTA are listed in Supplementary file.

### Attributes with potential impact on market access outcome

A list of attributes (variables) with potential impact on market access outcome was developed (Table II). These attributes were discussed and validated by an external expert group composed of four academic experts in pricing and market access for the USA and Europe, with an academic background in clinical pharmacology (USA and France), epidemiology (UK), and methodology (Germany).

**Table II:** Parameters evaluated for correlation of price and reimbursement outcome in repurposed drugs

Attribute	Type of variable	Description of variable	Geographical specificity
**Disease-related**			
ICD-10 chapter change	QUAL	Yes: Different ICD-10 chapter No: Same ICD-10 chapter	Non-country/continent specific
Prevalence change	QUANT	Difference between prevalence (for the USA and for the EU) of target and source product, by 100,000 population	Continent specific
DALY change	QUANT	Difference between DALY (for the USA and for the EU) of target and source product	Continent specific
Demographic group change	QUAL	Adults: From adults plus pediatrics to adults only No change: No change in demographic group Pediatrics: From adults only to adults plus pediatrics	Non-country/continent specific
Number of alternative treatments change	QUANT	Difference between the number of alternative treatments for the target and the source product	Non-country/continent specific
**Drug-related**			
Frequency of use change	QUAL	Increase: Increase in frequency of use No change: No change in frequency of use Decrease: Decrease in frequency of use	Non-country/continent specific
Change in administration setting	QUAL	Ambulatory: From hospital to ambulatory No change: No change in administration setting Hospital: From ambulatory to hospital	Non-country/continent specific
Addressing unmet needs	QUAL	Yes: Addresses an unmet need No: No change	Non-country/continent specific
Improved patient convenience	QUAL	Yes: More convenient use No: No change	Non-country/continent specific
**Repurposing-related**			
Repositioning	QUAL	Yes/No As per definition in Section I^1^	Non-country/continent specific
Reformulation	QUAL	Yes/No As per definition in Section I^2^	Non-country/continent specific
Repositioning target	QUAL	On-target: Target product acts via same pathway and/or protein interaction Off-target: Target product based on a newly discovered (or previously unexplored) pharmacological mechanism	Non-country/continent specific
Repositioning approach	QUAL	Serendipity: Fortuitous discovery of target product indication Hypothesis driven: Discovery of the new indication relies on understanding of the disease physiopathology and/or drug mechanism	Non-country/continent specific
Reformulation group	QUAL	Group 0: Chiral switch, other Group 1: Modified release formulations Group 2: New pharmaceutical form Group 3: New administration route	Non-country/continent specific
**Regulatory-related**			
Patent expiry	QUAL	Before: Repurposing occurred before patent expiry of source product After: Repurposing occurred after patent expiry of source product	Non-country/continent specific
Brand name	QUAL	Same: Target product developed under the same brand name Different: Target product developed under different brand names	Non-country/continent specific
Company	QUAL	Same: Target and source product developed by the same company Different: Target and source product developed by different companies	Non-country/continent specific
Approval time	QUAL	Before 1999 1999–2008 After 2008	Country specific
Designation change	QUAL	Orphan to orphan: From orphan to orphan Non-orphan: From orphan to non-orphan Orphan: From non-orphan to orphan No change: From non-orphan to non-orphan	Continent specific

1 Repositioning was defined as a process of finding for a known product, a new indication in a different therapeutic area, and/or via a different pharmacological target.

2 Reformulation was defined as a process of making a change into the formulation of a known product excluding dose changes and modifications involving a change in the structure of the active pharmaceutical ingredient.

The attributes were classified into four different groups and were analyzed according to their relation with disease, drug, repurposing strategy, or regulatory aspects. Table II presents the list of attributes, as well as the approach used for the analyses of the change introduced by the target product versus the source product.

#### Disease-related attributes

##### ICD-10 chapter change

Therapeutic indications for source and target products were retrieved from publicly available summaries of product characteristics (SmPCs) included in national regulatory data sources (Supplementary file). The corresponding ICD-10 code was retrieved for all case studies (6). Due to the multitude of ICD codes for all of case studies, it was decided to evaluate only for the change in ICD chapter, which was evaluated as a dichotomous variable.

##### Prevalence change

Prevalence information of the disease for which source and target products were indicated was extracted, to the extent possible, within the same data sources and methodological reports. Among the main sources for prevalence data were World Health Organization (WHO) health statistics data on prevalence (7, 8) (using *WHO Region A: Americas* as a proxy for the USA cases and *WHO Region B: Europe* for the European cases), a commercial database for epidemiological data—PatientBase^®^ (9), and the Centre for Disease Control and Prevention database (10) for the USA data that was unavailable from the previous sources. In situations where no prevalence data could be found for the studied indication due to a partial indication or subpopulation, we opted for the use of the disease as a whole as a proxy, referring to the ICD coding research. Prevalence change was assessed as a quantitative attribute (difference between prevalence for target and source product).

##### DALY change

Disability data were sourced from WHO's Global Burden of Disease Health Statistics database (11). The disability information collected was measured in standard disability adjusted life years (DALYs) weighted for age and using a 3% discount factor, as per the description of WHO's methodology (11, 12). DALY change was assessed as a quantitative attribute (the difference between DALYs for target and source product).

##### Demographic group change

Demographic group was described using the following population groups: pediatrics (<18 years old) and adults (≥18 years old). The change assessed whether the demographic group was modified.

##### Number of alternative treatments change

Alternative treatments were retrieved by searching for the indication in the Micromedex database (13). The change in number of alternative treatments was described as a quantitative variable, as well as the number of alternatives for the source product.

#### Drug-related attributes

All drug-related attributes were derived from the products’ SmPC.

##### Frequency of use

Change in frequency of use was coded in three categories (‘Increase,’ ‘No change,’ and ‘Decrease’) depending on the difference for source and target product.

##### Change in administration setting

Change in administration setting was coded in three categories: a change from hospital to ambulatory, from ambulatory to hospital, or no change.

##### Addressing unmet needs

An independent expert panel assessed this parameter. For example, situations where the target product was developed to treat a rare condition or for which few therapeutic alternatives existed at the repurposing time were considered as addressing an unmet need. Three criteria were used to define unmet need: alternative treatment goal, response rate, and safety/tolerability:

1. If alternative treatments were only symptomatic, then unmet need
2. If the response rate for alternative treatment was<75% in severe diseases, then unmet need
3. If the response rate for alternative treatment was<50% in less severe diseases, then unmet need
4. If the new treatment avoided severe safety or tolerability issues, then unmet need

This definition was achieved through a deliberative process, leading to a consensus among all experts.

##### Improved patient convenience

Better convenience of use with the target drug, in terms of mode of administration, was also evaluated by an independent expert panel. For example, formulations allowing a less frequent administration were considered as improving the patient convenience.

#### Repurposing-related attributes

All case studies were classified by repurposing strategy: repositioning, reformulation, and combinations according to the proposed definitions (1).

Repositioning cases were then categorized according to the pharmacological target (‘on-target’: target product effective in another disease via the same pathway or protein interaction as the source product; ‘off-target’: target product effective in a new or previously unexplored pharmacological mechanism from the source product) and the repositioning approach (‘serendipity’: repositioning by chance or random finding; ‘hypothesis-driven’: repositioning through a structured rational approach) (14). Reformulation case studies were categorized by reformulation groups 0, 1, 2, or 3, as detailed in the first article of this series, namely (1):

1. Group 0: Chiral switch, excipient change without pharmacokinetic impact and cases where none of the other classification of groups (1, 2, or 3) was applicable
2. Group 1: Same pharmaceutical form, same administration route, and different pharmacokinetic parameters (e.g., modified release formulations)
3. Group 2: Different pharmaceutical form, same or similar administration route, and same pharmacokinetic parameters
4. Group 3: Different pharmaceutical form and different administration route

#### Regulatory-related attributes

Regulatory attributes ‘approval time’ (before 1999, 1999–2008, after 2008) and ‘designation change’ (orphan to orphan, orphan, non-orphan, no change) were also included in the analyses (3).

Other regulatory attributes were also included: whether the repurposing was approved before or after patent expiry of the source product and if the target product had a change in the company or in the brand name compared with the source product.

### Statistical analysis

The different attributes were described as well as distributions of price changes according to all attributes to detect for possible trends and taking into account all merged repositioning and reformulation cases. Bivariate regression models were conducted to identify factors associated with price change. When the attribute was quantitative, the attribute value for the source product was introduced in the model, in addition to the attribute value for the target product, as another adjustment.

A similar analysis was then performed for reformulation and repositioning cases in the USA and in Europe, separately.

Attributes analyzed as quantitative data were presented as mean and standard deviation, and attributes analyzed as qualitative data were presented as proportions. A significance level of 5% was used for all analysis. Data were processed and analyzed using SAS 9.3 software (SAS Institute, Cary, NC, USA).

## Results

In this study, the 144 repurposing case studies that were used in the second article for assessment of regulatory implications were evaluated for mean price change. Out of the 144 repurposing cases, 74 were reformulations (26 in the USA and 48 in Europe), 76 were repositioning cases (23 in the USA and 53 in Europe), and 4 were combinations (1 in the USA and 3 in Europe). The total is not equal to 144 because 9 cases were both repositioned and reformulated including one case that was also combined. Three combination cases were excluded from the analysis due to the small number of cases, leading to a total of 141 reformulation and repositioning cases. Overall, 150 repurposing cases were considered in this study, taking into account the duplicate repositioned and reformulated cases.

Out of the 141 cases, the mean prices for 6 cases were found to be outliers (mean price change>400%) and were thought to be susceptible to bias of the statistical analysis. These outliers, not included in the analysis, are presented in Table III.

**Table III:** Outlier case studies for mean price change between source and target product

Drug	Repurposing type	Source (indication/pharmaceutical form)	Target (indication/pharmaceutical form)	Country	Price change	Price per therapeutic dose
Cetirizine	Reformulation	Rhinitis Racemic cetirizine	Rhinitis Enantiomeric switch: Levocetirizine	USA	+505%	Source: $0.39 Target: $2.37
Amiodarone chlorhydrate	Reformulation	Severe rhythm disorders	Severe rhythm disorders	FR	+614%	Source: F4.24 Target: F30.28
		Tablets	Solution for injection	UK	+1,032%	Source: €0.5357 Target: €6.0666
				DE	+1,730%	Source: €1.309 Target: €23.956
Esomeprazole	Reformulation	Gastro-esophageal reflux disorder	Gastro-esophageal reflux disorder	USA	+421%	Source: $4.37 Target: $22.77
		Tablets	Powder for solution for IV injection			
Finasteride	Repurposing	Benign prostatic Hyperplasia	Male pattern baldness Film-coated tablets	UK	+868%	Source: £0.0871 Target: £0.84357
		Film-coated tablets		DE	+476%	Source: €0.237 Target: €1.37
Retinoic acid/tretinoin	Reformulation+repurposing	Acute promyelocytic leukemia	Acne Topical gel	USA	+5,780%	Source: $2.72 Target: $159.95
		Capsules		UK	+13,900%	Source: £0.08 Target: £11.24
Sildenafil citrate	Reformulation	Pulmonary arterial hypertension	Pulmonary arterial hypertension	USA	+552%	Source: $0.75 Target: $9.80
		Tablets	Solution for injection	UK	+496%	Source: £0.2075 Target: £2.47625

We observed that all cases (n=144) were approved for reimbursement, which we considered as a proxy for a successful market access outcome conjointly with the mean price change. As such, we decided to use the mean price change analysis to determine the association of the different drug repurposing attributes and successful pricing outcome.

### Evaluation of the impact of attributes in mean price change

Table IV includes the results from the data extraction of each attribute and the mean price change. The decision on the methodology for analysis of the attributes was made after the data collection step: it was decided to analyze the attributes through a bivariate process because the number of cases in each individual attribute per region (USA or Europe) did not allow for a multivariate analysis.

**Table IV:** Impact of attributes in mean price change in repurposing: overall attributes

		Mean (SD)/freq (%) of attribute		Mean price change*	
					
Attribute	Category	N=141	N	Mean (SD)/correlation	P
**Geographical scope**					
Continent	Europe USA	96 (68%) 45 (31%)	57 31	0.50 (0.70) 0.47 (0.64)	0.81
**Disease-related**					
ICD-10 chapter change	Yes No	58 (40.28%) 83 (59.72%)	31 57	0.46 (0.64) 0.51 (0.70)	0.84
Prevalence change		1 (1,620)	70	−0.03	0.62**
DALY change		−9,90,088 (31,99,851)	54	0.07	0.08**
Demographic group affected	Adults No change Pediatrics	20 (14%) 114 (81%) 7 (5%)	7 77 4	0.28 (0.69) 0.52 (0.69) 0.25 (0.50)	0.27
Number of alternative treatments change		−5 (12)	67	−0.02	0.84**
**Drug-related**					
Frequency of use change	Increase No change Decrease	11 (8%) 95 (67%) 35 (29%)	2 61 25	−0.03 (0.05) 0.52 (0.67) 0.47 (0.71)	0.9
Change in administration setting	Ambulatory No change Hospital	– 131 (93%) 10 (7%)	– 87 1	– 0.46 (0.58) 3.74 (–)	<0.0001
Addressing unmet needs	Yes No	59 (42%) 82 (58%)	36 52	0.69 (0.74) 0.36 (0.60)	0.02
Improved patient convenience	Yes No	35 (25%) 106 (75%)	25 63	0.46 (0.59) 0.51 (0.71)	0.74
**Repurposing-related**					
Repositioning	Yes No	76 (54%) 65 (46%)	25 63	0.56 (0.59) 0.41 (0.78)	0.32
Reformulation	Yes No	74 (52.5%) 67 (47.5%)	42 46	0.36 (0.79) 0.61 (0.54)	0.07
Reformulation group	Group 0 Group 1 Group 2 Group 3	21 (28%) 13 (18%) 18 (24%) 22 (30%)	11 10 14 7	0.06 (0.44) 0.32 (0.69) 0.21 (0.45) 1.17 (1.33)	0.001
Repositioning (approach)	Serendipity Hypothesis driven	26 (34%) 50 (66%)	15 34	0.74 (0.68) 0.47 (0.54)	0.26
Repositioning (target)	On-target Off-target	65 (86%) 11 (14%)	43 6	0.54 (0.60) 0.66 (0.53)	0.57
**Regulatory-related**					
Patent expiry	Before After	113 (80%) 28 (20%)	80 8	0.51 (0.68) 0.32 (0.72)	0.44
Brand name	Same Different	77 (55%) 64 (45%)	46 42	0.65 (0.51) 0.32 (0.80)	0.02
Company	Same Different	117 (83%) 24 (17%)	78 10	0.50 (0.68) 0.44 (0.69)	0.78
Approval time	Before 1999 1999–2008 After 2008	37 (27%) 69 (50%) 31 (23%)	18 45 25	0.58 (0.73) 0.50 (0.72) 0.42 (0.58)	0.44
Designation change	Orphan to orphan Non-orphan Orphan No change	15 (11%) 2 (1%) 21 (13%) 103 (73%)	6 2 19 61	0.10 (0.47) 0.02 (0.03) 0.66 (0.48) 0.50 (0.74)	0.26

* Mean price change expressed as a quantitative variable. For example, a value of 0.50 should be interpreted as an increase in price by 50%.

** P value derived from linear regression with the baseline variable as a covariate.

An evaluation of the impact of each attribute on the mean price change of reformulations and repositioning was conducted for the USA and Europe separately, and the results are described in Table V and VI, respectively. The number of cases for which data was simultaneously available for source and target product for each attribute was limited, and frequently the number of paired cases with pricing data was lower than the total number of cases in each attribute. The individual results by country/repurposed type are therefore to be considered with caution.

**Table V:** Impact of attributes in mean price change: reformulations

		USA			Europe		
			
		Description of attribute	Description of mean price change* (%)		Description of attribute	Description of mean price change* (%)	
							
Attribute	Category	Mean (SD)/freq (%) of attribute	Mean (SD)/correlation	P	Mean (SD)/freq (%) of attribute	Mean (SD)/correlation	P
**Disease-related**							
ICD-10 chapter change	Yes No	4 (15%) 22 (85%)	−0.21 (0.84) 0.33 (0.53)	0.19	5 (10%) 43 (90%)	−0.65 (–) 0.44 (0.85)	0.2
Prevalence change		199 (1,014)	−0.56	0.03	0	0	–
DALY change		163,420 (783,733)	−0.56	0.04	0	0	–
Demographic group change	Adults No change Pediatrics	4 (15%) 21 (81%) 1 (4%)	0.09 (0.42) 0.27 (0.63) (–)	0.3	9 (19%) 37 (77%) 2 (4%)	0.54 (1.07) 0.39 (0.86) –	0.76
Number of alternative treatments change		−3.69 (12)	−0.09	0.82	−4 (13)	0.23	0.19
**Drug-related**							
Frequency of use change	Increase No change Decrease	2 (8%) 15 (58%) 9 (34%)	(–) 0.28 (0.43) 0.19 (0.81)	0.78	5 (10.5%) 29 (60.5%) 14 (29%)	– 0.27 (0.99) 0.59 (0.63)	0.29
Change in administration setting	Ambulatory No change Hospital	0 (0%) 23 (88%) 3 (12%)	– 0.24 (0.58) –	–	0 (0%) 41 (85.5%) 7 (14.5%)	– 0.29 (0.59) 3.74 (–)	<0.0001
Addressing unmet needs	Yes No	8 (31%) 18 (69%)	0.59 (0.83) 0.17 (0.56)	0.33	20 (42%) 28 (58%)	0.84 (1.35) 0.25 (0.56)	0.08
Improved patient convenience	Yes No	12 (46%) 14 (54%)	0.47 (0.63) 0.01 (0.48)	0.14	23 (48%) 25 (52%)	0.45 (0.59) 0.33 (1.22)	0.69
**Repurposing-related**							
Reformulation group	Group 0 Group 1 Group 2 Group 3	7 (27%) 6 (23%) 7 (27%) 6 (23%)	0.35 (0.22) −0.01 (0.68) 0.17 (0.61) 0.78 (0.55)	0.36	14 (30%) 7 (14.5%) 11 (23%) 16 (33%)	−0.01 (0.46) 0.54 (0.66) 0.23 (0.40) 1.33 (1.56)	0.01
**Regulatory-related**							
Patent expiry	Before After	17 (65%) 9 (35%)	0.36 (0.54) 0.02 (0.68)	0.31	37 (88%) 11 (23%)	0.40 (0.88) 0.50 (0.81)	0.84
Brand name	Same Different	8 (31%) 18 (69%)	1.12 (0.07) 0.07 (0.46)	0.004	21 (44%) 27 (56%)	0.50 (0.60) 0.34 (1.03)	0.6
Company	Same Different	18 (69%) 8 (31%)	0.24 (0.56) 0.24 (0.72)	0.99	37 (77%) 11 (23%)	0.46 (0.86) −0.29 (0.50)	0.22
Approval time	Before 1999 1999–2008 After 2008	7 (27%) 15 (58%) 4 (15%)	0.39 (–) 0.37 (0.55) −0.40 (0.57)	0.11	17 (35%) 23 (47%) 8 (16.67)	0.27 (0.66) 0.61 (1.04) 0.07 (0.46)	0.90
Designation change	Orphan to orphan Non-orphan Orphan No change	0 (0%) 0 (0%) 2 (8%) 24 (92%)	– – – 0.24 (0.58)	–	6 (12.5%) – – 42 (87.5%)	−0.19 (0.11) – – 0.47 (0.88)	0.18

* Mean price change expressed as a quantitative variable. E.g. a value of 0.50 should be interpreted as an increase in price by 50%.

** P value derived from linear regression with the baseline variable as a covariate.

**Table VI:** Impact of attributes in mean price change: repositionings

		USA			Europe		
			
		Description of attribute	Description of mean price change* (%)		Description of attribute	Description of mean price change* (%)	
							
Attribute	Category	Mean (SD)/freq (%) of attribute	Mean (SD)/correlation	P	Mean (SD)/freq (%) of attribute	Mean (SD)/correlation	P
**Disease-related**							
ICD-10 chapter change	Yes No	17 (74%) 6 (26%)	0.48 (0.80) 0.67 (0.52)	0.58	41 (77%) 12 (23%)	0.45 (0.55) 0.75 (0.45)	0.1
Prevalence change		78 (3,224)	−0.25	0.39**	−34 (2,013)	0.13	0.54**
DALY change		−30,39,602 (49,67,661)	−0.35	0.01**	−31,90,669 (53,42,501)	0.55	0.08**
Demographic group change	Adults No change Pediatrics	3 (13%) 18 (78%) 2 (9%)	0.23 (0.22) 0.56 (0.76) 1.00 (–)	0.67	8 (15%) 40 (75.5%) 5 (9.5%)	−0.29 (0.51) 0.68 (0.47) 0.00 (0.00)	0.0006
Number of alternative treatments change		−10 (17)	−0.14	0.63**	−10.5 (17)	−0.05	0.04**
**Drug-related**							
Frequency of use change	Increase No change Decrease	2 (10%) 16 (69%) 5 (23%)	(–) 0.62 (0.47) 0.45 (1.30)	0.91	5 (9.5%) 39 (73.5%) 9 (17%)	−0.07 (–) 0.65 (0.52) 0.11 (0.31)	0.30
Change in administration setting	Ambulatory No change Hospital	– 22 (96%) 1 (4%)	– 0.55 (0.70) –	–	– 51 (96%) 2 (4%)	(–) 0.56 (0.53) (–)	–
Addressing unmet needs	Yes No	8 (35%) 15 (65%)	0.62 (0.47) 0.49 (0.85)	0.69	26 (49%) 27 (51%)	0.66 (0.48) 0.42 (0.59)	0.20
Improved patient convenience	Yes No	1 (4%) 22 (96%)	– 0.55 (0.70)	–	– 53 (100%)	– 0.56 (0.53)	–
**Repurposing-related**							
Repositioning approach	Serendipity Hypothesis driven	9 (39%) 14 (61%)	0.72 (0.77) 0.39 (0.65)	0.31	17 (32%) 36 (68%)	0.76 (0.62) 0.50 (0.50)	0.23
Repositioning target	On-target Off-target	20 (87%) 3 (13%)	0.55 (0.73) 0.50 (0.71)	0.91	45 (85%) 8 (15%)	0.54 (0.54) 0.73 (0.53)	0.47
**Regulatory-related**							
Patent expiry	Before After	16 (70%) 7 (30%)	0.61 (0.65) 0.10 (1.27)	0.31	47 (89%) 6 (11%)	0.56 (0.53) (–)	–
Brand name	Same Different	14 (61%) 9 (39%)	0.56 (0.50) 0.52 (1.04)	0.89	34 (64%) 19 (36%)	0.75 (0.44) 0.24 (0.53)	0.004
Company	Same Different	17 (74%) 6 (26%)	0.62 (0.67) 0.20 (0.92)	0.32	45 (85%) 8 (15%)	0.56 (0.50) 0.59 (0.82)	0.90
Designation change	Orphan to orphan Non-orphan Orphan No change	3 (13%) 2 (9%) 5 (22%) 13 (56%)	0.54 (0.65) 0.02 (0.03) 0.77 (0.47) 0.57 (0.87)	<0.001	6 (11%) – 15 (28%) 32 (60%)	0.08 (–) – 0.63 (0.50) 0.53 (0.58)	0.81

* Mean price change expressed as a quantitative variable. E.g. a value of 0.50 should be interpreted as an increase in price by 50%.

** P value derived from linear regression with the baseline variable as a covariate.

#### Disease-related attributes

For USA repositioning, a DALY decrease for target product was associated with a mean price decrease when compared with the source product (p<0.05). Contrarily, a DALY increase for target product was associated with a mean price decrease for USA reformulations (p<0.05). The overall number of paired cases for DALY assessment was small (7 and 8 cases for USA/Europe repositioning and 6 cases for USA reformulations).

A significant impact on mean price change was found for repositioning cases in Europe where a change from ‘adults and pediatrics’ to ‘adults’ led to a 29% decrease in price, ‘no change’ led to a 68% increase, and a move from ‘adults’ to ‘adults and pediatrics’ did not impact price.

The mean change of number of alternative treatments was −5 in the overall analysis, suggesting a higher number of alternative treatments for the source compared to the target products. For that same attribute the mean price change was small but significant for Europe repositionings, with a small (5%) mean price decrease for the target product (p<0.05), in line with a decrease of number of therapeutic alternatives for the target product.

#### Drug-related attributes

Change in administration setting to hospital led to an overall mean increase of 374% (p<0.0001) for all repurposed cases; however, this result was driven by only 1 case of ‘change to hospital setting’ for reformulations. A significant increase in mean price was also seen for reformulations in Europe due to change to hospital setting. Total number of cases retrieved was small (41 cases with ‘no change’ and 1 case of ‘change to hospital setting’ for reformulations, and 73 cases with ‘no change’ for repositionings), so these results have to be considered with caution.

Overall, 42% repurposed of cases addressed an unmet need with a correspondent significant increase in price of 69%.

Improving patient convenience was more frequent for reformulations (46% in the USA and 48% in Europe were evaluated as improving patient convenience) than for repositionings (4% in the USA and no cases in Europe were assessed as improving patient convenience), with only 25% of overall cases presenting this attribute. There was no significant impact on price with this attribute.

#### Repurposing-related attributes

There was an even distribution within all cases in terms of type of reformulations, with reformulations in Group 3 (change in administration route) leading to a significantly higher price increase (p=0.001). This trend was also significant for European reformulation cases (p=0.01).

#### Regulatory-related attributes

An approval before patent expiry was reported for the vast majority of overall repurposing cases (80%), and this trend was similar for all reformulation and repurposing cases, in Europe and in the USA. No significant impact on price was reported.

Having the same brand name had a significant impact on price increase for the overall repurposing cases, as well in the separate analysis for reformulations in the USA and for repositionings in Europe.

Designation change to orphan drug had a significant impact on price increase for repositionings in the USA. Most of the repurposing cases (reformulations and repositionings pooled) had no change in designation, and reformulations had a lower ratio of designation change than repositionings.

## Discussion

The main objective of this article was to identify the determinants for successful market access outcome for repurposed drugs, measured by the mean price change, in order to allow a prediction of market access impact when pursuing the different repurposing strategies. It was assumed that reimbursement was a proxy for market access conjointly with price level approved. Consequently no further collection of data regarding regional listing or formulary inclusion for each case per country was collected. Nonetheless, it is possible that asymmetries in access due to those listings could exist for the source and target product.

We have selected several potential attributes that could be related to a change in price. It is noteworthy that we were limited by the availability of the data and had to collect information from various sources. Most information was retrieved from HTA reports and from other reliable public and proprietary databases. However, several variables were sometimes missing, and they were collected elsewhere as often as possible.

More than 60% of the reformulation and repositioning cases used for analysis were approved in Europe. This higher ratio of European case studies could have an impact on the interpretation of the aggregated repurposing case study results in Table IV and could affect the interpretation of each attribute weight to the price outcome because pricing and reimbursement environment and regulations are different in Europe than in the USA.

Prevalence of disease was considered an important attribute for evaluation. During data extraction of prevalence information, it was found that this information was not fully available or that conflicting data existed for many indications. It was then decided to choose the closest available indication for which disease prevalence data could be found as a proxy, using the information retrieved from the ICD coding research (e.g., ‘Parkinson's disease’ prevalence was used for the indication of ‘adjunctive treatment to levodopa in Parkinson's disease’ in the Parlodel^®^ case). The interpretation of prevalence impact on price outcome was still expected to be possible and fair. Differences in prevalence between source and target products’ indications were found to be minimal; hence, the result of negligible impact on price change was expected.

Mortality rate was initially considered to be an important attribute to be included in this analysis; however, data for the different cases was of poor quality, and it was decided to exclude this variable. We considered that DALYs associated with the disease could be considered as a partial proxy for mortality because they are a composite of years of life lost (YLL) due to premature mortality and years lost due to disability (YLD).

There seems to be no clear correlation between increase in disease severity (positive DALY change) and price increase: although for USA repositioning, a decrease in severity for the target product was associated with a significant price decrease, an increase in DALYs for reformulation was observed associated with a price decrease. A change in the therapeutic indication (evaluated by proxy through change in ICD-10 chapter) was only seen in 40% of cases, which can limit the change observed in DALYs. The number of paired cases for which DALY data was retrieved was also small.

When no change occurred in the demographics attribute, the price was also not changed, except for repositioning in Europe where ‘no change in demographics’ was associated with a significant price increase and a change to ‘adults’ was associated with a price decrease. These results in Europe could indicate a lower willingness to pay for repositioned products that, unlike their source product, do not address the pediatric population. However, due to the low number of cases, caution should be exercised in the interpretation of this attribute's impact on price.

Initially, we considered including the therapeutic goal of the repurposed product as an attribute (i.e., curative or palliative). However, we found that this attribute could be ambiguous and arbitrary, and we have decided not to include it in the analysis. In some situations, the drug could be used for both purposes (oncology drugs). The exact definition of cure in many diseases was not consensual; and the treatment goal could depend on the patient's health status and history of treatment.

It was initially considered that the number of pharmacological therapies available for a particular indication could have an impact on mean price change because it could allow for price comparisons among available therapies. However, when observing the study results, it seems that despite a lower number of therapeutic alternatives for the target product, there was no price change, except for Europe repositioning where there was a small but significant price decrease despite repositioning targets having fewer therapeutic alternatives. These results may be due to the already high number of alternatives at baseline for the source products, which makes a decrease of five therapeutic alternatives overall (or even 10.5 for repositionings in Europe) non-impactful for Payers' decision on price.

A significant price increase was observed for repurposed case studies when the target administration setting was changed from ambulatory to hospital only; however, this result needs to be interpreted carefully as only one case having this situation was observed.

Addressing unmet needs was also an attribute that lead to a significant price increase for all repurposed cases. This result is in line with the initial assumption that a product able to meet specific unmet needs is more likely to be granted a price premium, regardless of the repurposing strategy. Interestingly, despite this result, most of the repurposed cases were not able to be classified by our expert panel as addressing an unmet need.

The majority of reformulations were not able to be classified as drugs that lead to improved convenience. Because most of repositioning also did not improve convenience, it was not unexpected that the analysis of the pooled repurposed cases (presented in Table IV) also did not show an impact on price change.

Attributes related to the repurposed strategy used were included in the analysis in order to verify if there was a direct association between the individual repurposing type and the strategy used. From these attributes, only ‘being a reformulation with a change in administration route’ (Group 3) had a significant impact on price increase. In most cases where a change in administration route occurred, it was toward a more complex administration in a hospital setting.

Regulatory attributes were also included for the purpose of the statistical analysis: repurposing with ‘similar brand name as the source product’ had a significant impact on overall price increase.

An overwhelming number of repurposed cases (80%) were approved before patent expiry. This attribute did not impact the price change, which could be explained by the requirements for all drugs to demonstrate added value to regulators and payers, irrespectively of the patent status of the source product.

A designation change to orphan drug seemed to have a significant positive impact on price for repositioned products but only in the USA. These results suggest that willingness to pay for an orphan drug could be higher in the USA.

Our evaluation has some limitations—notably, the limited number of repurposed cases available for the statistical analysis. This may have reduced the ability of the statistical analyses to detect significant differences, although it was strongly expected. The number of cases was considerably reduced when we separated and created four groups for analysis: for repositioning and reformulation cases for the USA and Europe (Tables V and VI). Also, it would have been relevant to run a multivariate model in order to highlight trends, with adjustment on covariates. Nevertheless, the amount of cases with simultaneous source and target products’ paired-attribute and pricing data was not sufficient to provide reliable estimates. In the same way, it could have been relevant to run the models on reformulations and repositioning cases separately. This would require reproducing the analysis in several following years, when more data would be available. Third, the absence or low number of cases for the types of repurposing (such as combinations or repurposing aided by reformulation) did not allow verifying which pricing and market access trends were associated with those repurposing strategies.

All attributes considered for the statistical analysis were determined by the authors and were validated by an external expert group in order to ascertain that all attributes captured were relevant and that additional relevant attributes would not be missing from the analysis (to the extent of data availability). However, it should be considered that the expert panel expressed their own view points, and we cannot exclude that another panel could have suggested additional attributes or definitions.

Some of the non-quantitative attributes considered could have also benefited from having a validation by the same external expert panel (‘address unmet needs’ and ‘improving patient convenience’ attributes). Similarly, it cannot be excluded that the use of a different panel could have led to different classifications for these attributes.

Because this research was a comparative assessment, it was not possible to include cases for which the original drug was not approved/priced or when the repurposed drug was not approved/priced. This excluded the possibility of analyzing differences in price outcome of the so-called ‘shelved-recovered’ products, in which development for the initial indication was abandoned and later on was recovered for development in a different indication.

Finally, it is possible that the list of case studies for repurposed drugs included in this series was not exhaustive, despite the thorough literature review conducted. However, we considered that our methodology allows replication and a continuity of this research by other authors, and it can be considered to be unbiased in the cases collected due to public, and in most cases peer-reviewed, availability of the data.

## Conclusion

In this research, we aimed to determine which variables could predict a positive outcome from price change, a proxy for market access outcome in this study. Due to the complexity inherent to pricing decisions, multiple variables (attributes) were selected and analyzed. We found that different variables could have a different impact on pricing outcome depending on the type of repurposing strategy. The results of our study indicated that the variables that impacted a positive price change for repurposed drugs were the ability of the repurposed product to address unmet needs and to be a reformulation where the target product had a different and more complex administration route as well as having a similar brand name for the source and target products. Although the number of paired data available to evaluate each variables’ impact on price change was in several situations limited, our results suggested that orphan designation had also a positive impact for repositioning in the USA. Although a change of administration from ambulatory to hospital setting seemed to be significantly correlated with a price increase for the target product, only one case was retrieved for this parameter; as such, it was not possible to infer a firm correlation between this parameter and a change in price.

In order to establish a multifactorial association between variables and pricing outcome for the different repurposing strategies, it would be valuable to continue to collect further repurposed cases for future research.

## Supplementary Material

Drug repurposing in pharmaceutical industry and its impact on market access: market access implications
