# Supplementary material for: Drug repurposing in pharmaceutical industry and its impact on market access: market access implications
Source: J Mark Access Health Policy. 2014 Feb 25;2:10.3402/jmahp.v2.22814. doi: 10.3402/jmahp.v2.22814 (PMC4865758; doi:10.3402/jmahp.v2.22814)
Supplement: Drug repurposing in pharmaceutical industry and its impact on market access: market access implications [file JMAHP-2-22814-s001.docx]

## **Supplemental 1. Price data sources**

| **Scope** | **Source** | **Link** |
| --- | --- | --- |
| Global | ZenRx | <http://www.zenrx.org/> |
| Europe | PharmOnline International | <http://www.ihs.com/products/global-insight/industry-analysis/healthcare-pharmaceutical/pharma-pricing-data.aspx> |
| France | Amélie, Assurance maladie - Base des Médicaments et Informations Tarifaires | <http://www.codage.ext.cnamts.fr/codif/bdm_it/index_presentation.php?p_site> |
|  | Ministry of Health | <http://medicprix.sante.gouv.fr/welcome.do> |
|  | E-Vidal | <http://www.evidal.net/index.php> |
| Germany | Lauer Taxe | <http://www2.lauer-fischer.de/produkte/arzneimitteldaten-online/webapor-lauer-taxe/> |
|  | Gelbe Liste | <http://www.gelbe-liste.de/home> |
| UK | NHS Business Services Authority (Drug Tariff) | <http://www.nhsbsa.nhs.uk/PrescriptionServices/924.aspx> |
|  | British National Formulary | <http://www.bnf.org/bnf/index.htm> |
|  | MIMS online | http://www.mims.co.uk/ |
| USA | Medi-Span® Price Rx ® | <http://www.medispan.com/drug-pricing-analysis-pricerx.aspx> |

## **Supplemental 2. Reimbursement and HTA data sources**

| **Scope** | **Source** | **Link** |
| --- | --- | --- |
| Europe | PharmOnline International | <http://www.ihs.com/products/global-insight/industry-analysis/healthcare-pharmaceutical/pharma-pricing-data.aspx> |
| France | HAS | <http://www.has-sante.fr/> |
|  | Amélie, Assurance maladie - Base des Médicaments et Informations Tarifaires | <http://www.codage.ext.cnamts.fr/codif/bdm_it/index_presentation.php?p_site> |
|  | Thériaque | <http://www.theriaque.org/apps/recherche/rch_simple.php> |
|  | Commission de Transparence | http://www.has-sante.fr/portail/jcms/c_6056/fr/recherche-avancee?expression=exact&expression=exact&text=Saisir+vos+mots+cl%C3%A9s&text=Saisir+vos+mots+cl%C3%A9s&liaison_word=and&searchOn=fullText&catMode=or&dateMiseEnLigne=indexDateFrom&dateDebut=&dateFin=&typesf=opinions%2Fgenerated.AVISMedicament&search_antidot=&portlet=c_39085&sort=pdate&replies=50 |
| Germany | Lauer Taxe | <http://www2.lauer-fischer.de/produkte/arzneimitteldaten-online/webapor-lauer-taxe/> |
|  | German DIMDI reimbursement Prices | <http://www.dimdi.de/static/en/amg/fbag/index.htm> |
|  | GKV-Spitzenverband | <http://www.dimdi.de/dynamic/de/amg/fbag/downloadcenter/2013/januar/01-15/> |
| UK | British National Formulary (BNF) | <http://www.bnf.org/bnf/index.htm> |
|  | National Institute for Health and Care Excellence (NICE) | <http://www.nice.org.uk/> |
|  | Scottish Medicines Consortium (SMC) | [http://www.scottishmedicines.org.uk/SMC_Advice/Advice_Directory/SMC_Advice_Directory (UK launch date)](http://www.scottishmedicines.org.uk/SMC_Advice/Advice_Directory/SMC_Advice_Directory) |
| USA | Medicare Part D: Reference Formulary | <http://www.cms.gov/apps/frf/license.asp?file=/PrescriptionDrugCovContra/downloads/FormularyReferenceFile.zip> |

## **Supplemental 3. Sources for Regulatory and Market approval history**

| **Scope** | **Source** | **Link** |
| --- | --- | --- |
| EU | The European Medicines Agency (EMEA) | <http://www.ema.europa.eu/ema/index.jsp?curl=pages/medicines/landing/epar_search.jsp&mid=WC0b01ac058001d124> |
| France | Thériaque | <http://www.theriaque.org/apps/recherche/rch_simple.php> |
|  | Haute Autorité de Santé (HAS) | <http://www.has-sante.fr/> |
|  | E-Vidal | <http://www.evidal.net/index.php> |
| Germany | FachInfo | <http://www.fachinfo.de/> |
|  | G-BA | <http://www.g-ba.de/> |
| UK | EMC | <http://www.medicines.org.uk/emc/> |
|  | National Institute for Health and Care Excellence (NICE) | <http://www.nice.org.uk/> |
|  | Scottish Medicines Consortium (SMC) | [http://www.scottishmedicines.org.uk/SMC_Advice/Advice_Directory/SMC_Advice_Directory (UK launch date)](http://www.scottishmedicines.org.uk/SMC_Advice/Advice_Directory/SMC_Advice_Directory) |
| USA | Drugs@FDA | <http://www.accessdata.fda.gov/scripts/cder/drugsatfda/index.cfm> |
